# Structure prediction of porous organic crystals

**DOI:** 10.1039/d5ra09332e

**Published:** 2026-02-04

**Authors:** Musiha Mahfuza Mukta, Romain Perriot, Shinnosuke Hattori, Wei Zhou, Qiang Zhu

**Affiliations:** a Department of Mechanical Engineering and Engineering Science Charlotte NC USA qzhu8@charlotte.edu; b Theoretical Division, Los Alamos National Laboratory Los Alamos NM 87545 USA; c Advanced Research Laboratory, Research Platform, Sony Group Corporation 4-14-1 Asahi-cho, Atsugi-shi 243-0014 Japan shinnosuke.hattori@sony.com; d NIST Center for Neutron Research, National Institute of Standards and Technology Gaithersburg MD 20899-6102 USA wzhou@nist.gov; e North Carolina Battery Complexity, Autonomous Vehicle and Electrification (BATT CAVE) Research Center Charlotte NC 28223 USA

## Abstract

In this work, we explore the possibility of applying automated crystal structure prediction to reproduce the experimentally identified metastable porous polymorphs. Using our recently developed High-Throughput Organic Crystal Structure Prediction (
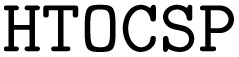
) framework, we conducted a systematic study on five representative organic crystalline systems including hydrogen-bonded frameworks (HOFs), featured by the presence of significant porosity, in conjunction with different choices of energy models from classical, machine learning force fields, tight binding to density functional theory. Our results suggest that the current structure generation framework, with careful selection of symmetry conditions, is likely to generate rather complex and abundant metastable crystal candidates for porous crystals. In conjunction with the recent advance in universal machine learning force fields, it becomes possible to identify experimental structures as the energetically favorable candidates from a simple energy *versus* density analysis, thus paving the way for computational design of complex porous materials with the target systems prior to the experimental synthesis and characterization.

## Abstract 2

In this work, we explore the possibility of applying automated crystal structure prediction to reproduce the experimentally identified metastable porous polymorphs.

## Graphical abstract

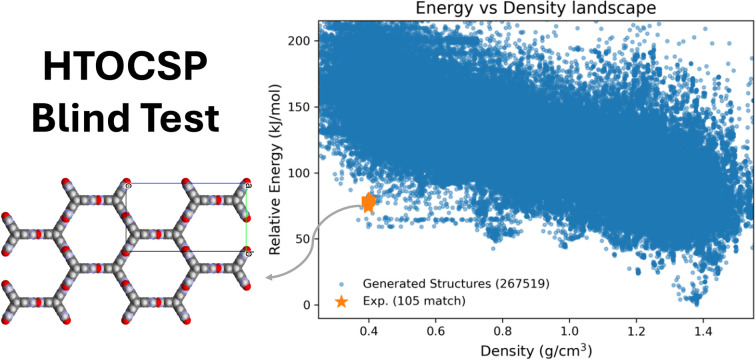

## 1 Introduction

In recent years, high-throughput computational screening of organic crystals has become a viable strategy for designing and searching new materials with improved physical properties.^1–6^ However, most studies have focused on experimentally resolved structures drawn from existing databases, such as the Cambridge Structural Database (CSD)^7^ and the Crystallography Open Database (COD).^8^ In practice, the ability to screen likely crystal packings prospectively—prior to synthesis and characterization—would be highly valuable.^9,10^

Over the past two decades, crystal structure prediction (CSP) for small organic molecules has advanced rapidly.^11–17^ In a typical CSP study, the objective is to generate a tractable set of low-energy (stable or metastable) crystal packings that are experimentally plausible, using efficient exploration algorithms.^10,18,19^ Recently, we introduced the High-Throughput Organic Crystal Structure Prediction (
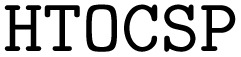
) framework, an open-source platform that automates CSP workflows from minimal molecular input by integrating existing toolkits for force-field assignment, structure generation and energy ranking.^20^ Accordingly, we demonstrated its performance across diverse molecular systems, with an emphasis on recovering dense packing motifs. As expected from earlier studies,^10^ thermodynamic ground states overwhelmingly correspond to densely packed arrangements. For many application domains (*e.g.*, organic semiconductors and pharmaceuticals), focusing on dense forms is often sufficient.

In practice, however, there also exist many other applications that would benefit from metastable crystal forms, including low-density porous polymorphs that can exhibit desirable properties such as selective gas adsorption, molecular recognition, and catalytic activity. Porous organic crystals, constructed through weak intermolecular interactions (such as hydrogen bonds and van der Waals interactions), represent an emerging class of crystalline materials in which permanent porosity arises from the ordered packing of discrete organic molecules rather than from extended coordination or covalent networks. Within this family, hydrogen-bonded organic frameworks (HOFs) have attracted particular attention as a unique platform for designing lightweight, solution-processable porous solids with tunable functionality.^21^ Early molecular-tectonics studies established that rationally designed hydrogen-bond donors and acceptors can assemble into robust three-dimensional porous networks with remarkable structural integrity, even through extensive guest–exchange cycles.^22^ More recent HOFs, such as the flexible microporous framework HOF-5, exhibit substantial permanent porosity, guest-responsive lattice expansion/contraction, and selective gas sorption behavior, highlighting the rich structure–property landscape accessible through judicious hydrogen-bond design.^23^ At the same time, the weak yet partly directional nature of hydrogen bonding makes HOF packing highly sensitive to molecular geometry and conformation; consequently, even small structural variations can lead to distinct polymorphs with different pore architectures and properties.

From the CSP perspective, predicting porous organic crystals is inherently more challenging due to two main factors. First, the potential structural space for porous crystals is significantly larger than that of dense packings. The presence of large voids and channels leads to a rugged energy landscape with numerous local minima corresponding to different molecular orientations with similar energies. Consequently, the configurational space requiring exploration is substantially expanded. Second, practical applications often favor metastable polymorphs with low densities rather than the thermodynamically stable dense-packed ground state. This creates additional challenges in identifying which candidate structures among a large pool of generated metastable forms are most likely to be realized experimentally. While several CSP studies have addressed porous organic crystals,^24–26^ this area remains largely underexplored.

In this work, we aim to explore the capability of the 
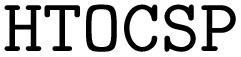
 framework to predict porous organic crystals, building upon our earlier work on dense packings^20^ and complementing previous CSP studies of porous molecular crystals.^24–26^ We begin by presenting the selection criteria for our benchmark systems and providing an overview of the 
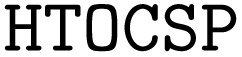
 workflow. Subsequently, we evaluate these systems using complementary structure-sampling strategies combined with alternative energy models (including classical force fields, machine-learning potentials, and semi-empirical methods), and assess their effectiveness in recovering low-density polymorphs. We conclude with a discussion of new directions for improving accuracy in future studies.

## 2 Systems of choices

**Fig. 1:**
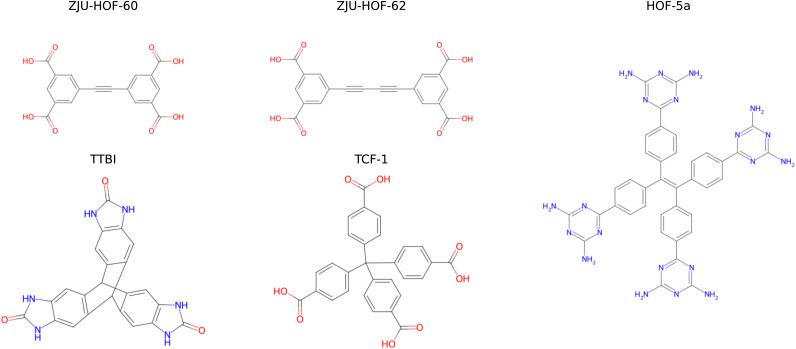
The selected five molecular systems in the present work.

Fig. 1 displays the five porous organic crystalline systems selected for this study. These systems were chosen to represent a diverse range of structural complexity, pore architectures, and hydrogen-bonding motifs characteristic of HOFs:

• ZJU-HOF-60 and ZJU-HOF-62 are two recently reported HOFs, based on 5,5′-(1,2-ethynediyl)bis(1,3-benzene-dicarboxylic acid) and 5,5′-(1,3-butadiyne-1,4-diyl)bis(1,3-benzene-dicarboxylic acid) molecular units, respectively.^26^ Due to their relatively simple planar molecular geometry, both HOFs have 2D layered structures, in which the molecules are connected through hydrogen bonds within individual layer and the interlayer stacking is enabled by π–π and van der Waals interactions. They exhibit great potential for certain hydrocarbon separation applications, thanks to their 1D channel-like pores along with the dialkynyl and carboxylic sites for selective gas adsorption.

• TTBI is a triptycene trisbenzimidazolone-based HOF that exhibits a three-dimensional porous framework stabilized by extensive hydrogen-bonding networks. The rigid triptycene scaffold enforces specific molecular orientations that facilitate permanent porosity.^24^ Notably, TTBI has been reported to exhibit multiple polymorphic forms (α-, β-, and γ-phases) with varying pore sizes and shapes, making it an ideal candidate for studying the relationship between molecular design, packing motifs, and porosity in HOFs. Additionally, this system was explored in a previous CSP study,^27^ providing a valuable benchmark for comparison with our CSP methodology.

• TCF-1 is a tetracarboxylic-based HOF, obtained by crystallization of methanetetrabenzoic acid.^25^ Two structural forms have been reported. The first is called “porous-TCF-1”, which has moderate porosity and exhibits structural flexibility, characterized by reversible expansion and contraction of the pores upon guest adsorption and removal. The second form is a nonporous phase, called “dense-TCF-1”. The existence of both porous and dense polymorphs of this HOF presents a great opportunity for us to test our predictive capability as well.

• HOF-5a is a well-known prototypical flexible HOF, constructed from 4,4′,4″,4‴-*tetra*(2,4-diamino-1,3,5-triazin-6-yl)tetraphenylethene.^23^ It exhibits moderately high porosity and interesting gas adsorption/separation properties. Upon guest inclusion, its channel-like pore can expand its cross-section, leading to a significant increase in pore volume, up to 70%. The bulk size and the intrinsic flexibility of the molecular building unit make this HOF a particularly challenging benchmark system.

In selecting these systems, we aimed to cover a spectrum of HOF architectures, from rigid to flexible frameworks, and from simple layered to complex hydrogen-bonding motifs, as well as varying degrees of porosity. This diversity allows us to systematically evaluate the performance of our CSP framework across different structural challenges inherent to porous organic crystals. As shown in Table 1, it is worth noting that most of the structures have been reported to adopt the structures with fractional *Z*′ numbers, due to the presence of high molecular symmetry. In practical CSP applications, it is often necessary to convert these structures into equivalent subgroup representations with integer *Z*′ numbers to facilitate structure generation and sampling.

**Table 1:** Crystallographic data and porosity values of the eight porous organic crystals studied

System	Symmetry	*Z*′	Cell parameters (Å; °)	Density (g cm^−3^)	Porosity
ZJU-HOF-60 (ref. 26)	*I*2/*m*	1/4	*a* = 3.65, *b* = 16.44, *c* = 20.95, *β* = 91.6	0.937	46.5%
ZJU-HOF-62 (ref. 26)	*P*3_1_	1	*a* = 16.46, *c* = 10.10	0.795	52.8%
α-TTBI^27^	*P*4_2_/*m*	1/2	*a* = 22.51, *c* = 7.34	0.755	59.8%
β-TTBI^24^	*P*1̄	1	*a* = 7.25, *b* = 13.03, *c* = 20.66, *α* = 72.5, *β* = 86.3, *γ* = 74.0	0.782	55.4%
γ-TTBI^27^	*P*6_3_/*mmc*	1/12	*a* = 23.22, *c* = 7.29	0.412	79.0%
Porous-TCF-1 (ref. 25)	*P*4_2_/*n*	1/4	*a* = 13.10, *c* = 8.08	1.239	20.6%
Dense-TCF-1 (ref. 25)	*I*4̄	1/4	*a* = 12.53, *c* = 7.53	1.394	1.2%
HOF-5a^23^	*C*2/*m*	1/4	*a* = 14.35, *b* = 17.87, *c* = 12.26, *β* = 121.7	0.954	41.1%

## 3 Computational methodology

To predict porous organic crystals, we employed the 
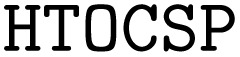
 framework,^20^ which integrates various tools for structure generation, optimization, and analysis. Below, we outline the key components of our computational methodology.

### 3.1 The HTOCSP workflow

In the 
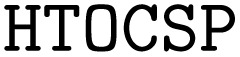
 framework, it uses the list of chemical SMILES strings as the input to generate the 3D molecular structure using 
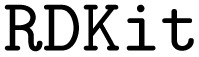
.^28^ The generated molecule is then used to create trial crystal structures usin
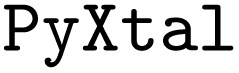
,^29^ which allows for flexible specification of space groups, *Z*′, and other structural parameters, based on two common sampling strategies based on either the width-first search (WFS) or depth-first search (DFS).^20^ Each of the generated structures are subsequently optimized using the classical force field powered by the CHARMM code,^30^ and then more accurate machine learning force fields. Finally, the optimized structures are analyzed and ranked based on their relative stabilities and structural similarity to known polymorphs. For molecules with rotatable bonds, the initial structures are created with randomized torsional angles, so different conformations are sampled across the population. Each generated crystal is then fully relaxed under periodic boundary conditions, allowing both the lattice and all atomic coordinates, including intramolecular torsions to optimize. So, conformational changes are naturally driven by the crystal packing environment, without requiring a separate conformer library. However, PyXtal's default process is sampling-based, it samples conformers and uses tolerance rules (and compatibility with Wyckoff-site symmetry) to decide which orientations are acceptable. So, molecular flexibility is handled automatically during generation and relaxation.

To illustrate the usage of 
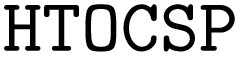
, we provide a sample script in Listing 1 that demonstrates how to set up and run a CSP calculation for the TTBI using the DFS sampling strategy with GAFF force field. The script includes parameter setup, and the sampling process. After running the simulation, it generates random structures, and from those one can check for the experimental one.
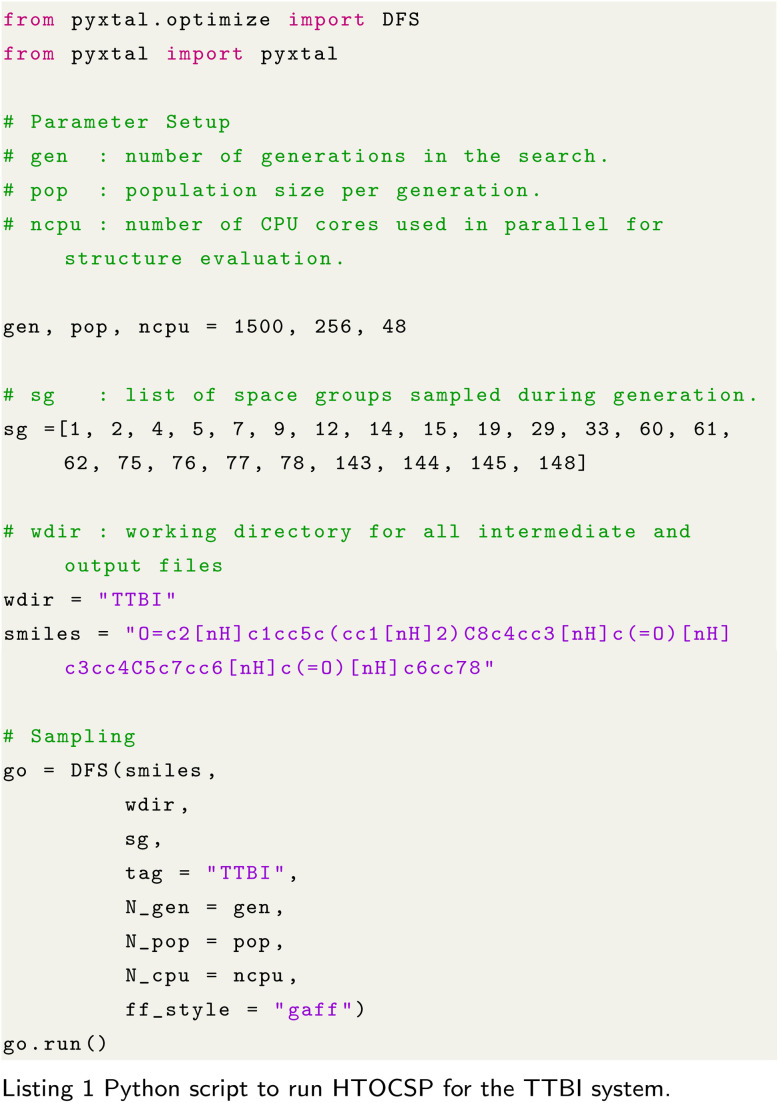


### 3.2 Structural sampling strategies

In this study, we focus on two strategies for generating trial structures by setting different space groups:

#### 3.2.1 Blind search on the known space group symmetries

This approach assumes prior knowledge of the space group. If the *Z*′ is fractional, we convert it to an equivalent subgroup representation with *Z*′ = 1 (corresponding to the case of molecules occupying the general Wyckoff position). The purpose of the test is to evaluate the efficiency of different sampling strategies and energy models in recovering the target structure within a limited number of sampled structures.

#### 3.2.2 Blind search on a list common space group with *Z*′ = 1

This approach mimics a more realistic CSP scenario where the space group is unknown. We select a list of 23 common space groups for organic crystals (*P*1, *P*1̄, *P*2_1_, *C*2, *Pc*, *Cc*, *P*2/*c*, *P*2_1_/*c*, *C*2/*c*, *P*2_1_2_1_2_1_, *Pna*2_1_, *Pca*2_1_, *Pbcn*, *Pbca*, *Pnma*, *P*4, *P*4_1_, *P*4_2_, *P*4_3_, *P*3, *P*3_1_, *P*3_2_ and *R*3̄) and set *Z*′ = 1 for all trials. The goal is to assess the likelihood of identifying the target structure within a limited number of sampled structures across multiple space groups.

To check if the target structure is found in our search, the StructureMatcher module in 
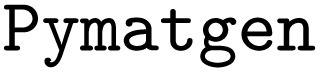
 (ref. 31) is used. In this module, we ignore all H atoms and build a one-to-one map between each molecule in the unit cell, then check the largest root mean squared error (RMSE) between each atomic pair. By default, two structures are considered identical if the fractional length tolerance is 0.25, the fractional site tolerance is less than 0.25, and the angle tolerance is less than 5°.

### 3.3 Energy model choices

After the structures are generated, they need to be ranked based on their relative stabilities. In the 
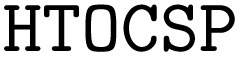
, we have implemented several energy models for geometry optimization and energy evaluation, including classical force fields (FFs), machine learning potentials (MLPs), semi-empirical tight binding (DFTB) methods, and density functional theory (DFT). In this work, we focus on the following energy models for CSP structure search.

#### 3.3.1 Classical force fields for geometry relaxation

We employ GAFF^32^ and OpenFF^33^ for fast lattice and molecular relaxations. GAFF is a widely used general Amber force field that provides reasonable accuracy for a broad range of organic molecules. OpenFF uses direct chemical perception (SMIRNOFF) and often improves transferability over GAFF. In the 
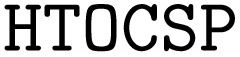
, parameters are assigned *via*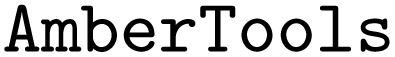 (ref. 34) for GAFF and the OpenFF Toolkit for OpenFF, and structures are minimized with 
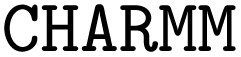
.^30^ Here, atomic partial charges are molecule-specific and consistent across the search.

#### 3.3.2 Machine-learning force fields for refinement

MACE^35^ is an equivariant message-passing neural network potential that achieves near-DFT accuracy at significantly reduced computational cost. We use MACE for post-optimization single-point energy evaluation and optional short local relaxations on GAFF/OpenFF-relaxed geometries to improve ranking accuracy at modest cost relative to DFT.

Additionally, we consider the following models for additional energy ranking of top candidates extracted from the CSP search.

##### 3.2.2.1 Alternative MLPs for post-optimization ranking

We also consider MACE-OFF,^36^ a MACE variant optimized for organic systems, and UMA,^37^ a universal potential trained on a broad corpus of molecules and materials with small, fast variants suitable for CSP.

##### 3.2.2.2 Semi-empirical tight binding

LATTE^38^ is a self-consistent charge transfer density functional tight-binding (SCC-DFTB) code, which provides a semi-empirical tight-binding Hamiltonian. SCC-DFTB^39,40^ can provide substantially better accuracy than classical FFs at a fraction of DFT cost. Here, we use the *lanl*31 parameterization developed for organic molecules,^41^ with additional pairwise dispersion corrections.^42^ Although the parameterization was fitted on small organic molecules in the gas phase, it has been demonstrated to provide an accurate description of organic crystals in the solid phase, reproducing lattice parameters and surface energies with good accuracy.^43^

##### 3.2.2.3 Density functional theory

DFT serves as the highest-accuracy stage for validating and final re-ranking of top candidates. All computations were performed using VASP^44^ 6.4.3, employing the GGA-PBE^45^ functional with PAW-PBE pseudopotentials. Long-range dispersion interactions were included *via* Grimme's DFT-D3 (ref. 46) correction with zero damping function (IVDW = 11), ensuring an accurate description of van der Waals forces. Geometry optimizations were carried out using the conjugate-gradient algorithm (IBRION = 2) with full relaxation of both atomic positions and lattice parameters (ISIF = 3).

Using these models, we performed additional full structure relaxation (including both atomic coordinates and cell parameters) to obtain the final energy ranking.

## 4 Results and discussions

### 4.1 Blind search with the known space group

**Fig. 2:**
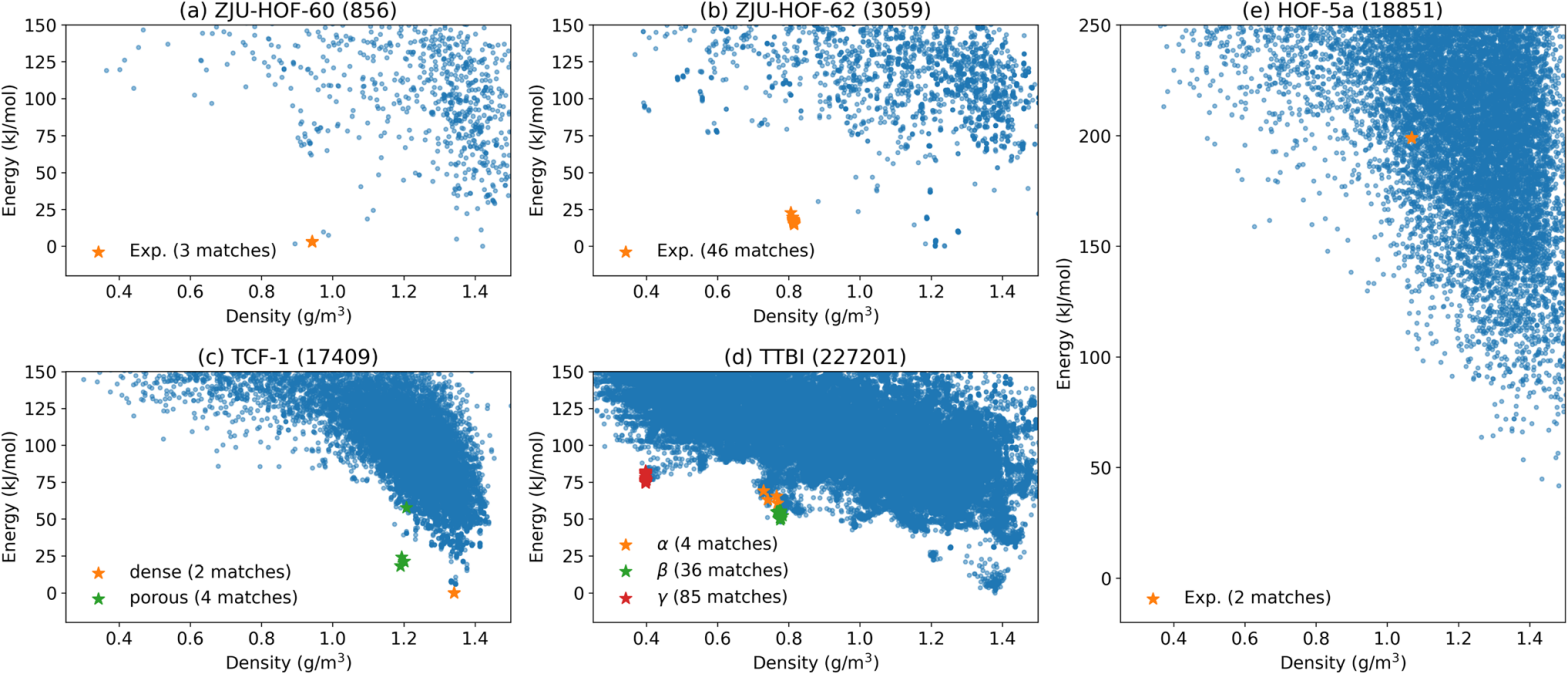
Structure search based on the known space group symmetry for the five systems: (a) ZJH-HOF-60, (b) ZJU-HOF-62, (c) TCF-1, (d) TTBI and (e) HOF-5a. In the title of each plot the number of sampled structures are denoted within the parentheses. Here, ‘matches’ denote the generated structures that are identified as equivalent to the target experimental structure.

Fig. 2 summarizes the results for the case when the target space group symmetries are known. For ZJU-HOF-60 (*I*2/*m*) and ZJU-HOF-62 (*P*3_1_), we attempted the structure search with *C*2 and *P*3_1_, respectively. As shown in Fig. 2a and b, both simulations return high structure matches rate of 4–10 per 1000 sampled structures. We also emphasize that GAFF force field tends to generate the incorrect ground state geometry for both ZJU-HOF-60 and ZJU-HOF-62, while OpenFF provides better description of the geometry. This highlights the importance of using the correct force field even for the initial stage of structure screening. For the polymorphic cases of TTBI and TCF-1 in Fig. 2c and d, we conducted CSP simulations with (*P*1̄, *P*4_2_, *P*2_1_2_1_2_1_) for TTBI and (*P*2_1_, *Pc*) for TCF-1, the prediction of different polymorphs requires more search efforts about 20–200k trial structures, but the cost are not significant since it is mostly about classical force field optimizations. For HOF-5a (see Fig. 2c), the simulation attempted at the space group *Cc*, returning a success rate of 1 per 10k structures that is similar to TCF-1.

In general, identifying the experimental structure is straightforward when the space group symmetry is known. Using MACE for energy ranking, all experimental structures fall within 0–20 kJ mol^−1^ above the computed ground state. Even the higher-energy TTBI polymorphs (Fig. 2d) appear as distinct local minima within the nearby low-density basin. The sole exception is HOF-5a: the matched structure lies about 200 kJ mol^−1^ above the global minimum and about 100 kJ mol^−1^ above the lowest minimum at a similar density. This large discrepancy underscores a current limitation of the present energy models and this will be discussed later.

### 4.2 Blind search with 23 most common space groups

We next considered a realistic scenario when the space group is unknown, in which we performed blind searches with *Z*′ = 1 over 23 common organic-crystal space groups. This setting is more challenging than the single known-space-group tests because (i) sampling capacity must be divided among symmetry classes and (ii) incorrect symmetry assumptions can delay convergence to the experimental packing. To improve efficiency, we employed the DFS strategy, in which each new trial selects a space group from the list and low-energy structures are preferentially retained and propagated.^20^ Consequently, the effective space-group visitation frequencies become non-uniform and biased toward symmetries that support lower-energy packings for the given molecule.

**Fig. 3:**
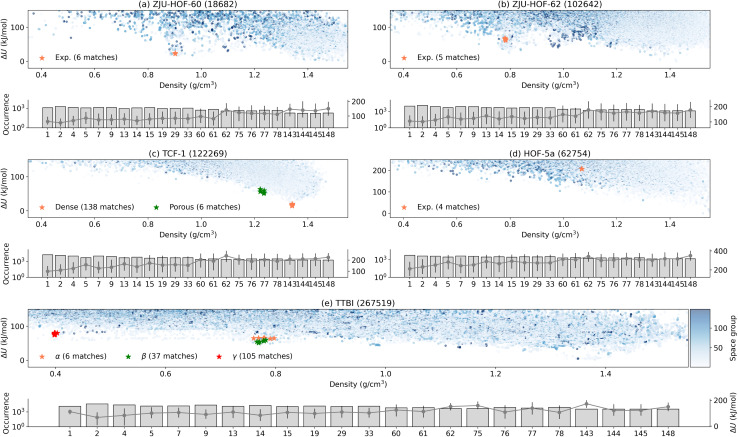
Structure search performed within the common space group symmetries for the five systems: (a) ZJU-HOF-60, (b) ZJU-HOF-62, (c) TCF-1, (d) HOF-5a and (e) TTBI. For each system, the upper panel displays a scatter plot of energy *versus* density colored by the space group number, and the lower panel shows the occurrence of structures in each space group. In case of lower panel, bar height (plotted against the left *y*-axis) indicates the number of generated structures; and gray circles connected by lines (plotted against the right *y*-axis) denote the mean relative energy Δ*U* within each space group, while vertical whiskers represent the energy spread (10–90th percentile).

Fig. 3 summarizes the aggregate outcomes. The overall search effort required to recover the experimental forms increased only modestly after introducing the multi–space-group pool. For example, the γ-TTBI polymorph (experimental *P*6_3_/*mmc*, 
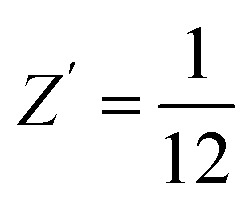
) can be represented in several subgroup settings with *Z*′ = 1 on a general Wyckoff position; it was matched 105 times in ∼2 × 10^5^ trials—comparable to the rate observed in the focused search of Fig. 2. Similar behavior was found for α-TTBI and β-TTBI: match frequencies remained within the same order of magnitude as their known-symmetry counterparts. Furthermore, dense-TCF-1 exhibited an even more favorable outcome, with match frequencies improving notably under common-space-group sampling, indicating that the DFS energy bias quickly suppresses unproductive symmetry branches while amplifying access to the correct packing basin. However, the strategy may favor the formation of low energy structures. For ZJU-HOF-60 and ZJU-HOF-62, the layered low-density motifs emerged early and repeatedly, despite competition from low-symmetry triclinic and monoclinic settings. Hence, it is fair to conclude that blind search for porous structure with fractional *Z*′ does not significantly increase the overhead as long as the true symmetry is included in the given list of space groups in the input.

A more significant challenge is the energy ranking. After search for more space groups, we have found that most of the target structures remain energetically favorable as either the global minimum or the local minimum in the surrounding density range. However, ZJU-HOF-62 in Fig. 3 is no longer favorable as compared to that Fig. 2. The case of HOF-5a is about the same. This imposes the additional challenge to identify the experimental structures if they do not appear as either the global or local energy minimum.

### 4.3 Energy ranking with different models

**Fig. 4:**
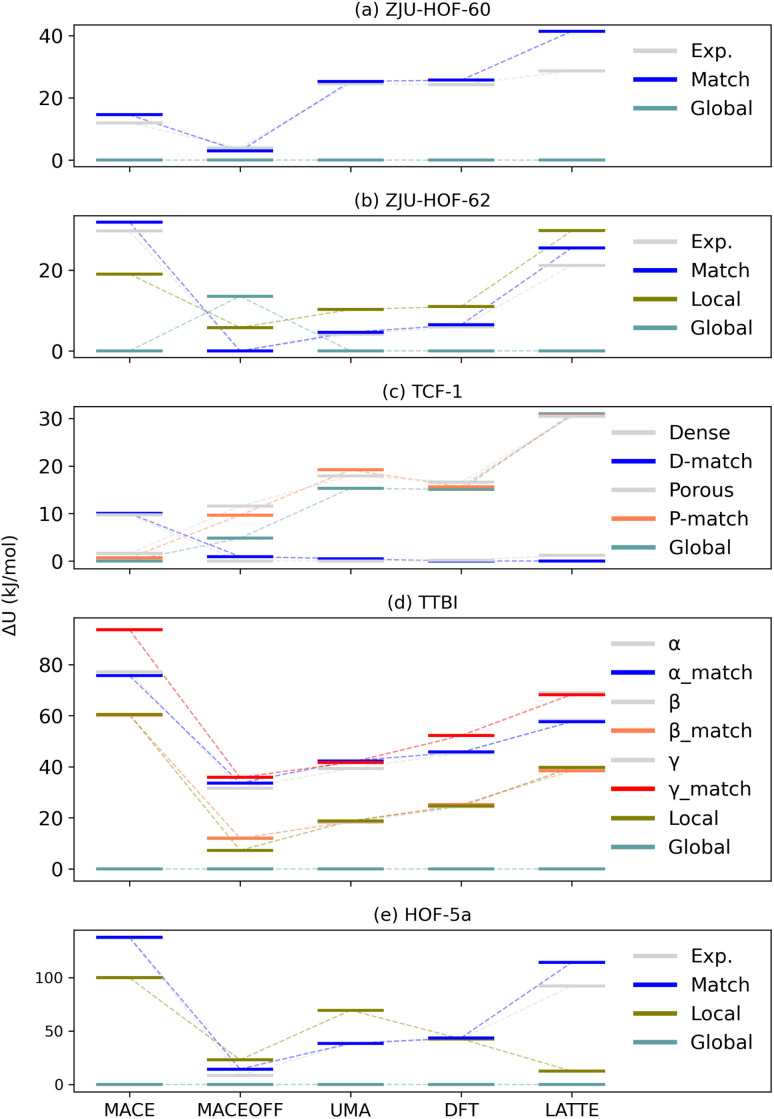
Energy ranking across different methods for multiple representative structures found in each system: (a) ZJU-HOF-60, (b) ZJU-HOF-62, (c) TCF-1, (d) TTBI and (e) HOF-5a. Here, ‘Global’ refers to the structure with the lowest overall energy among all candidates found in the search, while ‘Local’ refers to the lowest-energy structure within the specific density region corresponding to the experimental structure.

To address the energy-ranking challenges, we re-ranked representative structures (experimental form, matched instances, global minimum, and the nearest local minimum at comparable density) with MACE, MACE-OFF, UMA, LATTE, and DFT. The results are summarized in Fig. 4.

In general, MACE provides a reliable ordering for most systems at low cost as compared to the DFT results. MACE-OFF, while somewhat more expensive, did not consistently improve the rank correlation for the present porous sets—likely reflecting limited coverage of large low-density HOF topologies in its training data. LATTE (SCC-DFTB) is more costly than the ML single points yet still far cheaper than DFT; LATTE demonstrates an accuracy similar to MACE, and tends to overestimates the energy difference as compared do DFT. This comes as no surprise considering that HOF were not included in the development of the *lanl*31 parameterization used here.

Most importantly, UMA yields an ordering that is qualitatively consistent with the DFT re-ranking in all benchmark cases. Both UMA and DFT correct the MACE misordering for HOF-5a, stabilizing the experimental porous form relative to the spurious nearby low-density minimum. This improvement directly increases the likelihood of retaining the true polymorph during down-selection. Given its accuracy – cost balance, UMA is a practical choice for intermediate (post-FF) energy refinement in future large CSP screenings, reserving DFT for only a small final candidate set.

### 4.4 Improved energy ranking of HOF-5a using UMA

**Fig. 5:**
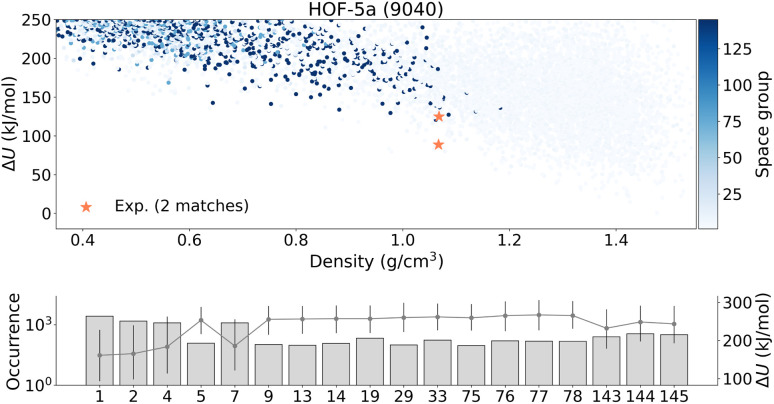
Structure search performed within the common space group symmetries for HOF-5a. The upper panel displays a scatter plot of energy *versus* density colored by the space group number, and the lower panel shows the occurrence of structures in each space group; where bar height indicates the number of generated structures; gray circles connected by lines denote the mean relative energy Δ*U* within each space group, and vertical whiskers represent the energy spread.

Using UMA as the energy model substantially improved the energy ranking of the generated HOF-5a candidates, getting the matched structure within a local minimum region of the energy landscape. Moreover, we observed a clear improvement in efficiency: 2 matches were found within 9040 generated structures, compared with 4 matches within 62 754 structures in our previous setup. In Fig. 5, the two matched structures initially appear at different energies, but after proper relaxation they converge to the same final structure and overlap with each other. This indicates that they correspond to the same minimum and therefore have the same relaxed energy; the initial energy difference is due to imperfect starting geometries and model noise. Although UMA is more computationally expensive, we recommend using it for complex systems where MACE struggles to rank candidates correctly and fails to place the matched structures in the metastable region.

## 5 Conclusions

We have demonstrated that automated crystal structure prediction of porous organic crystals is achievable using the 
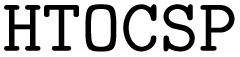
 framework, despite the inherent challenges posed by their expanded configurational space and energetic preference for metastable low-density forms. Through systematic evaluation of five benchmark systems, we established that carefully designed sampling strategies combined with multi-stage energy refinement can reliably recover experimentally observed porous polymorphs.

When the experimental space group is known, the 
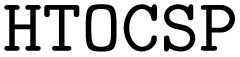
 sampling generates the target structures efficiently, with match rates substantially improved by short classical FF relaxations. However, in realistic blind-search scenarios across common space groups with *Z*′ = 1, the two-stage relaxation protocol of initial FF minimization followed by ML refinement—becomes essential for identifying true matches from the energy ranking perspective. In particular, UMA consistently rank experimental porous forms as energetically competitive within low-density basins, enabling effective post-screening to capture subtle dispersion and electrostatic balances. As such, CSP may be promising as a predictive tool for the rational design of functional porous organic materials prior to synthesis.

## Author contributions

S. H., W. Z. and Q. Z. proposed this idea and supervised this research. M. M. M. performed the majority of materials simulations. R. P. participated on the tight binding simulation and structural analysis. All coauthors designed the research, analyzed the calculations and wrote this manuscript.

## Conflicts of interest

There are no conflicts to declare.

## Data Availability

The data and scripts used in this study, are available in https://github.com/MaterSim/HTOCSP.
